# Quantitative and Qualitative Characterization of* Gentiana rigescens* Franch (Gentianaceae) on Different Parts and Cultivations Years by HPLC and FTIR Spectroscopy

**DOI:** 10.1155/2017/3194146

**Published:** 2017-06-01

**Authors:** Lu-Ming Qi, Ji Zhang, Yan-Li Zhao, Zhi-Tian Zuo, Hang Jin, Yuan-Zhong Wang

**Affiliations:** ^1^College of Traditional Chinese Medicine, Yunnan University of Traditional Chinese Medicine, Kunming 650500, China; ^2^Institute of Medicinal Plants, Yunnan Academy of Agricultural Sciences, Kunming 650200, China; ^3^Yunnan Technical Center for Quality of Chinese Materia Medica, Kunming 650200, China

## Abstract

*Gentiana rigescens* Franch (Gentianaceae) is a famous medicinal plant for treatments of rheumatism, convulsion, and jaundice. Comprehensive investigation of different parts and cultivation years of this plant has not yet been conducted. This study presents the quantitative and qualitative characterization of iridoid glycosides from* G. rigescens* performed by HPLC and FTIR spectroscopy techniques. The accumulations of loganic acid, swertiamarin, gentiopicroside, and sweroside were determined. Results indicated that their content and distribution in different parts and cultivation years exhibit great variations. Gentiopicroside was identified as the most abundant compound among iridoid glycosides and its highest level was observed in the root of 2-year-old plant. With respect to qualitative variation of metabolic profile, the 1800–800 cm^−1^ band of FTIR spectra successfully discriminated different parts and cultivation years with the aid of PLS-DA. In addition, combined with PLSR, the feasibility of FTIR spectroscopy for determination of gentiopicroside was investigated by selecting characteristic wavelengths (1800–800 cm^−1^), which presented a good performance with a residual predictive deviation (RPD) of 3.646. Our results suggested that HPLC and FTIR techniques can complement each other and could be simultaneously applied for comparing and analyzing different parts and cultivation years of* G. rigescens*.

## 1. Introduction

Nowadays, medicinal plants have occupied an important place in people's lives. As natural resources, they exert wide range of pharmacological and biological properties which can be responsible for primary health needs of humans [1]. As a consequence, these plants have been used to produce drugs or health foods which make a great contribution to the increase of economic interest [2]. However, compared with synthetic medicinal products, their qualities vary drastically on the basis of geographical origin, processing method, growth stage, and so forth [3–7].

Among above factors, the selections of medicinal parts and cultivation years are critical to the quality of medicinal plants. In general, a particular organ which has the highest accumulation of active compound is considered as the medicinal part, and other organs may be abandoned due to their low curative effects [8]. Also, a certain medicinal plant is always harvested in the selected cultivation age in modern agriculture for higher accumulations of bioactive metabolites [9]. In recent years, in order to detect the quality of medicinal plants, many studies have been carried out to understand the variation of chemical information caused by these factors [10, 11].

**Figure 1:**
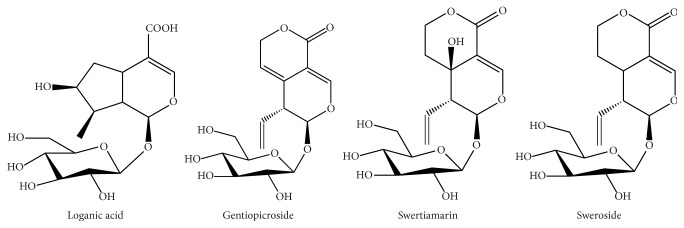
The chemical structures of biomarkers of* G. rigescens*.

*Gentiana rigescens* Franch (Gentianaceae) (see Supplementary Material available online at https://doi.org/10.1155/2017/3194146) which is cultivated in the Yunnan Province of China has been known as a famous medicinal plant for thousands of years [12]. Traditionally, the root is employed as the medicinal part for its curative properties. This plant has been recorded in Chinese Pharmacopoeia (2015 version) for treatments of rheumatism, convulsion, and jaundice [13]. Up to now, many reports have demonstrated that its health benefits are mainly related to iridoid glycosides, including gentiopicroside, swertiamarin, sweroside, and loganic acid (Figure 1) [14–17]. Especially, gentiopicroside has been regarded as the marker component of* G. rigescens* to evaluate its quality in Chinese Pharmacopoeia [13]. According to preliminary statistics, the demand is nearly 1000 tons per year for producing drugs or other health products due to extensive therapeutical effects [18]. Therefore, it has been cultivated on a large scale in the Yunnan Province to satisfy the wide applications.

As many medicinal products of* G. rigescens* appear in different formulations such as powders and capsules, it is unrealistic to identify them just by physical appearance and taste. Many analytical methods including spectroscopic and chromatographic techniques have developed to detect intrinsic quality of* G. rigescens*. For example, Zhao et al. [18] have applied successfully Fourier transform infrared (FTIR) spectroscopy to discriminate this plant from different geographical origins. High performance liquid chromatography (HPLC) has been carried out to measure the bioactive compounds in aerial parts by Shen et al. [19]. The chemical variations between raw and processed* G. rigescens* have also been described based on FTIR and ultra-fast liquid chromatography [20]. Moreover, ultraviolet spectroscopy [21] and micellar electrokinetic chromatography [22] methods have been used to characteristic information in previous studies. The tissue culture materials during different growing stages were also studied and compared by Pan et al. [12], demonstrating that major metabolites of different organs are diverse. However, a report related to their chemical characteristic of different parts and cultivation ages is still lacking.

HPLC is a quantifiable technique which can describe the variation of a particular chemical compound of samples accurately and specifically [23]. Comparatively, FTIR spectroscopy provides more detailed information, which can represent the overall metabolic profile of samples [24]. When combined to chemometrics, this technique is employed not only to classify samples but also to predict the content of specific compound by constructing different models [25–27]. In the present study, the chemical variations of different parts and cultivation ages of cultivated* G. rigescens* were characterized and compared based on HPLC and FTIR techniques. The feasibility of FTIR spectroscopy for determination of the marker component gentiopicroside was also investigated.

## 2. Experimental

### 2.1. Sample Preparation

In this experiment, 28 individuals containing 1-year-old (9 individuals), 2-year-old (9 individuals), and 3-year-old (10 individuals) plants were collected from Kunming cultivation base (Yunnan, China) and authenticated by Professor Hang Jin (Institute of Medicinal Plants, Yunnan Academy of Agricultural Sciences). After collection, they were manually washed and separated into different parts (roots, stems, leaves, and flowers). A total of 105 samples (27 roots, 27 stems, 28 leaves, and 23 flowers) were collected and they were dried in a drying oven at 50°C for 24 hours. After smash and sieving, the powder was stored for next analysis.

### 2.2. HPLC and FTIR Analysis

The chromatographic analysis was conducted with an Agilent 1260 Infinity System (Agilent Technologies, Palo Alto, USA) which consisted of a G1311C VL quaternary pump, a G1329B ALS autosampler, a G1316A thermostatted column compartment, and G1315D diode array detector. The column employed is an Agilent Zorbax AB-C18 column (5 *μ*m, 4.6 × 250 mm) and the mobile phase consisted of 0.1% formic acid (A) and acetonitrile (B) aqueous solution at a flow rate of 1 mL/min. The gradient program was set as follows: 0–5 min, 5% B; 5–10 min, 5%–10% B; 10–26 min, 10%–26% B; 26–30 min, 26%–30% B. The column temperature was controlled at 30°C. The injection volume of each sample was 10 *μ*L and the chromatogram was acquired at 241 nm wavelength.

A FTIR spectrometer (PerkinElmer, USA) equipped with a DTGS detector was applied for scanning samples. The spectra were recorded in the range of 4000–400 cm^−1^ with a resolution of 4 cm^−1^. For each spectrum, a total of 64 scans were performed. The powder of each sample (1.0 mg) and KBr (100.0 mg) were weighed precisely in an electronic balance (Precisa, Switzerland). They were mixed uniformly and pressed into a tablet using a tablet press (Shanghai Shanyue Instrument Inc., China). Subsequently, the tablet was scanned by FTIR spectrometer. Background absorptions of H_2_O and CO_2_ were deducted automatically. Each spectrum was acquired in triplicate and the average spectrum was selected for the next analysis.

### 2.3. Reagents

HPLC grade methanol was purchased from Xilong Chemical Co., Ltd. (Guangzhou, China) and analytical grade formic acid and acetonitrile were obtained from Sigma-Aldrich (USA), respectively. Deionized water used in the solutions and dilutions was prepared by an ultrapure water system (Millipore, USA). The chemical standards of loganic acid, swertiamarin, gentiopicroside, and sweroside were provided by Shifeng Biological Technology Co., Ltd. (Shanghai, China), and Chinese National Institute for Food and Drug Control (Beijing, China). KBr was purchased from Fengchuan Fine Chemical Research Institute (Tianjin, China).

### 2.4. Extraction of Iridoid Glycosides

The samples were extracted using 80% methanol according to the study by Shen et al. [19]. Each sample (25 mg) was accurately weighed in an electronic balance (Precisa, Switzerland) and then ultrasonically dissolved in 1.5 mL of 80% methanol for 40 min. Finally, additional solvent was transferred into volumetric flask to make up for volatilization of methanol. The obtained solutions were stored in darkness at 4°C and filtered through a 0.22 *μ*m membrane filter (Millipore, USA) before analysis by HPLC.

### 2.5. Data Analysis

Smoothing can eliminate noise signal in raw spectra introduced by external factors [28]. Baseline correction was essential for a good visual classification or prediction [29]. Derivation algorithm was used to remove the baseline shifts and overlap peaks [30]. Multiplicative scatter correction (MSC) can eliminate the noise signal caused by light scattering effect of particles [31].

Partial least square discriminant analysis (PLS-DA) is a widely used classification technique based on dimension reduction of original spectral data [32]. In the present paper, it was applied to present the two-dimensional visual classification according to different parts and cultivation years. Further, in order to describe the wave numbers that make higher contributions to the classifications, the loading plots of PC1 and PC2 are presented.

Partial least squares regression (PLSR) is a famous linear regression method to interpret the relationship between spectra data (*X*) and the response vector (*Y*) for quantitative analysis [33]. This technique has been commonly used to determine the active compounds of medicinal plants based on spectroscopic data [27]. In the present study, the characteristic band of 1800–600 cm^−1^ was selected to investigate the prediction ability of PLSR algorithm. The pretreatments of first derivative (FD), SD, and MSC were considered to optimize PLSR model. The performance of regression model was evaluated based on coefficient of determination (*R*^2^), root mean square error of cross validation (RMSECV), root mean square error of prediction (RMSEP), and residual predictive deviation (RPD) [34, 35].

The samples were divided into two groups by Kennard-Stone algorithm [36] consisting of a calibration set (70 samples) and a test set (35 samples). The PLS-DA and PLSR techniques were achieved using the software of SIMCA (Version 13.0, Umetrics, Umeå, Sweden).

The concentrations of loganic acid, swertiamarin, gentiopicroside, and sweroside were calculated by the similarity evaluation system for chromatographic fingerprint of traditional Chinese medicine (Version 2004a, Chinese Pharmacopoeia Commission, China). The statistical variations of total iridoid glycosides compounds concentration according to different parts and cultivation ages were compared using one-way analysis of variance. Differences were considered significant at a level of *p* ≤ 0.05.

## 3. Results and Discussions

### 3.1. Method Validation of HPLC Analysis

The calibration curves of loganic acid, swertiamarin, gentiopicroside, and sweroside were performed plotting the peak areas against the standard concentrations, respectively. A good correlation coefficient (*r*^2^) and linearity were obtained for each compound (Table 1). The limits of detection (LOD) and limits of quantitation (LOQ) of the investigated compounds were estimated by the response at signal-to-noise ratios (S/N) of 3 and 10, respectively. The calibration curves, *r*^2^, LOD, and LOQ of standard compounds are given in Table 1.

The precision was determined by analyzing five mixed sample solutions. The relative standard deviation (RSD) values of peak areas for the precision were <1.14. The stability was evaluated by analyzing the same sample solution at 0, 4, 8, 12, and 16 h, respectively. To confirm the repeatability of this method, five replicate analyses of mixed sample were analyzed. The RSDs of peak areas for repeatability and stability were in the range 1.23–2.36%. The recovery test was carried out by adding three different amounts (low, intermediate, and high spike) of the standard solution into mixed sample. The average recovery of each iridoids compound was 97.24–102.07%.

### 3.2. Variation of Iridoid Glycosides among Different Parts

**Figure 2:**
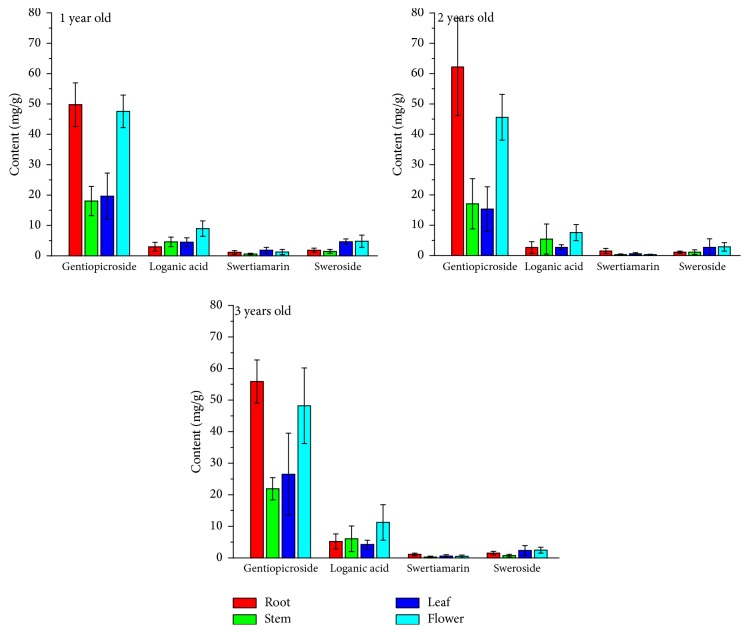
Quantitative variation of iridoid glycosides between different organs. (Description: this figure shows the quantitative variation of four iridoid glycosides between different organs of 1-, 2-, and 3-year-old* G. rigescens *resources based on HPLC method. The content of metabolites is represented as the average value and the standard deviations of these metabolites are also presented.)

The contents of iridoid glycosides (loganic acid, gentiopicroside, sweroside, and swertiamarin) in roots, stems, leaves, and flowers of* G. rigescens* are presented as the average values (Figure 2). It is observed that these compounds are commonly present in all plant parts. Regardless of cultivation age, the accumulation of gentiopicroside is found to be the highest, followed by loganic acid, and only tiny amounts of sweroside and swertiamarin are detected. As a consequence, the content of gentiopicroside has been emphasized in Chinese Pharmacopoeia [13].

Comparatively, root shows the highest syntheses of gentiopicroside, followed by flower while amounts in stem and leaf are relatively low. Maybe this result can provide the evidence why root is used as the main medicinal part all along the history. Remarkably, the level of gentiopicroside in flower is also high, which has a small difference from that in root. Previous research has been reported that the flower of* Gentiana macrophylla* has a high accumulation capacity for this compound [37]. Thereby, this part of* G. rigescens* can be regarded as a potential medicinal organ, to some degree.

With respect to the other compounds, their distribution regularities in different parts are variable. The highest level of loganic acid is observed in flower. The concentration of swertiamarin in these four parts is significantly low, the roots and the leaves being its more concentrated parts. For sweroside, the accumulations in leaf and flower are higher than that of the other parts. In light of the descriptions, the accumulations of these four compounds are variable obviously among different organs, which are in accordance with other medicinal plants [38, 39]. The selection of certain part of* G. rigescens *impacts the medicinal quality.

### 3.3. Variation of Iridoid Glycosides among Cultivation Ages

**Figure 3:**
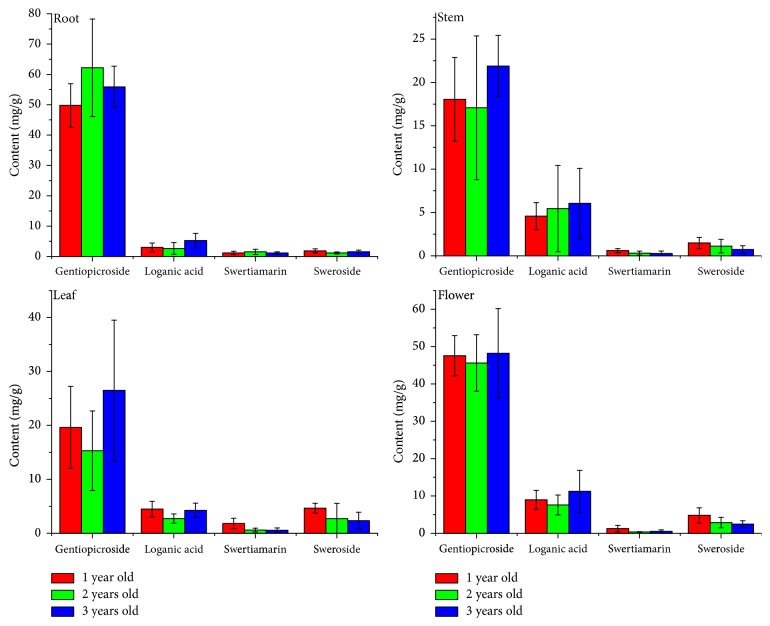
Quantitative variation of iridoid glycosides between cultivation ages. (Description: this figure shows the quantitative variation of iridoid glycosides between cultivation ages of different organs of* G. rigescens *resources based on HPLC method. The content of metabolites is represented as the average value.)

According to the study on tissue culture materials [12], the accumulation of iridoid glycosides in* G. rigescens* is different in various growth stages. Quantitative variations of iridoid glycosides among different cultivation ages are presented in Figure 3.

These compounds are dynamic during the growth of* G. rigescens*. The gentiopicroside of root is increased in the first two years while it subsequently decreased in the third year. The change regularities of this compound of other parts show an opposite trend. Loganic acid of root, leaf, and flower decreases at first and then increases. With respect to stem, the content of loganic acid grows steadily during three years. For swertiamarin and sweroside, the levels are gradually declined during the growth of this plant in stem, leaf, and flower. In root, the swertiamarin falls down after it ascends first and the sweroside shows an opposite change against swertiamarin during three years. It could be concluded that the levels of these compounds obviously changed with the change of cultivation ages. The highest amount of gentiopicroside is identified in the root of 2-year-old plant, which may have the best medicinal quality.

In addition, the variation of total iridoid glycosides compounds is displayed in Table 2. Obviously, the variation caused by different parts is more noticeable than that by different cultivation ages. Particularly, there are not significant differences of these compounds of stem and flower among different cultivation ages. In root, these compounds of 1-year-old samples are significantly different to that of 2-year-old samples. In leaf, these compounds of 1-year-old samples are significantly different to that of the others. For different parts, the total iridoid glycosides compounds of root and flower are significantly different to that of stem and leaf.

### 3.4. Discrimination of Different Cultivation Ages and Parts

**Figure 4:**
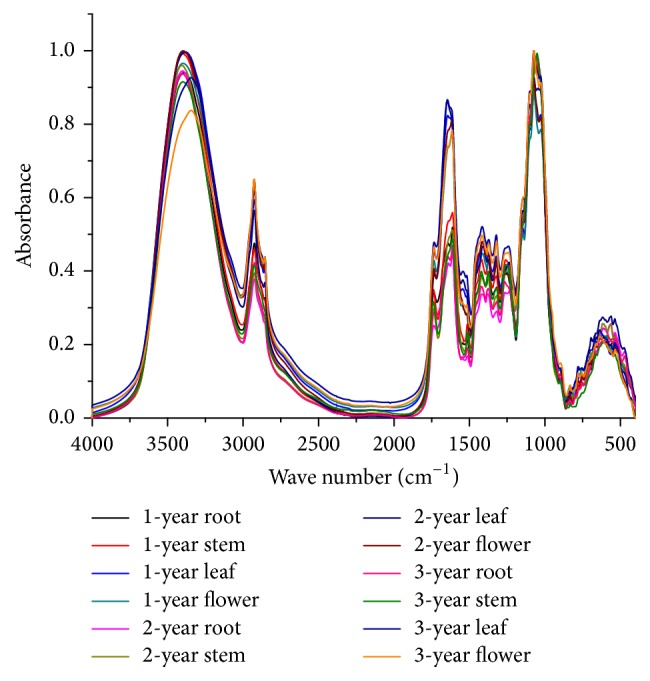
The original FTIR spectra of* G. rigescens *samples. (Description: this figure shows the original FTIR spectra of* G. rigescens *samples from different organs and cultivation ages. These spectra are exhibited as mean spectra.)

In the present paper, the original FTIR spectra of different parts and cultivation ages of* G. rigescens *are exhibited in Figure 4. It can be seen that these metabolic spectra share some similar characteristic bands concentrating on 1800–800 cm^−1^ region, such as the bands around 1735, 1611, 1375, 1268, and 1075 cm^−1^. The absorbance of characteristic peaks is different, which may make contributions to differentiate samples. However, these slight differences may be insufficient to distinguish samples only by simple visual inspection. Hence, the application of pretreatment methods and chemometrics may be useful to achieve the classification objective.

**Figure 5:**
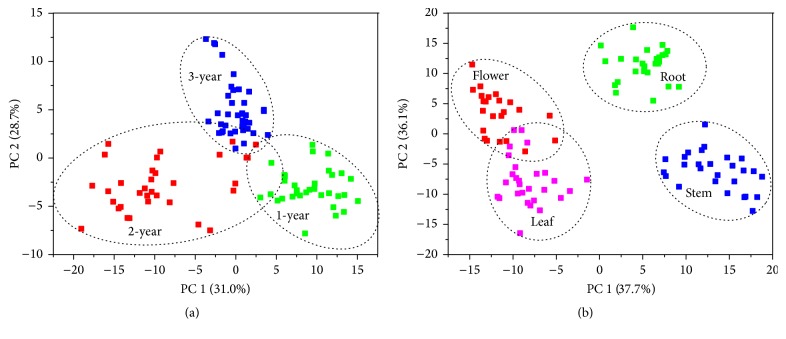
The score plots of* G. rigescens *samples based on the first two PCs. (Description: (a) shows the classification result between different cultivation ages, where the first 2 PCs can explain 59.7% information of total variance. (b) shows the classification result between different organs, where the first 2 PCs can explain 73.8% information of total variance.)

Second derivative (SD) was used to enlarge the differences between FTIR spectra [40]. The PLS-DA was used to classify samples by dimensionality reduction and visualize the samples' distribution in score plots. The 1800–800 cm^−1^ spectra region was used to identify samples among different cultivation ages and parts. The score plots based on the first two principal components (PCs) are presented in Figure 5.

Figure 5(a) exhibits the classification result among different cultivation ages. PC1 and PC2 can represent 59.7% information of total variance. The score plot presents a satisfactory separation except some overlaps between samples that are 2 and 3 years old.

Figure 5(b) shows the classification result of among different parts. PC1 and PC2 can explain 73.8% information of total variance. A good classification performance is displayed, which demonstrates the variation of metabolic profile among different parts. The samples of leaf and flower are closer in the plot, indicating that they could have a similar metabolic profile. The conclusion is in accordance with the study carried out by Pan et al. [41]. According to the total variance of PC1 and PC2, the chemical variations caused by different parts are more noticeable than that by different cultivation ages (73.8% > 59.7%). This result is corresponding to the conclusion of Table 2.

### 3.5. Loading Analysis of FTIR Spectra

**Figure 6:**
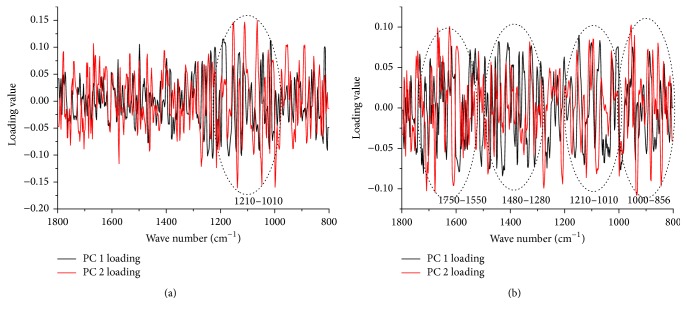
The loading plot of PC1 and PC2. (Description: (a) shows loading plot of the classification between different cultivation ages and (b) shows loading plot of the classification between different organs.)

According to the classification results, the first two PCs can achieve satisfactory clustering among different cultivation ages and parts.  Figure 6(a) shows loading plot of the classification between different cultivation ages. The band around 1210–1010 cm^−1^ has the most important influence on both PC1 and PC2 loadings. Yang et al. [42] have reported that this band may be mainly assigned as the stretch vibrations of C-O and C-OH bonds which are caused by terpene, glucoside, and phenols compounds.

Figure 6(b) presents the loading plot according to different organs. Besides the 1210–1010 cm^−1^ band, the bands around 1750–1550 cm^−1^, 1480–1280 cm^−1^, and 1000–856 cm^−1^ also make significant contributions to this classification. The peak around 1610 cm^−1^ represents an asymmetric stretch of C-C bond which may be assignable to terpene compounds. The vibration at 1453 cm^−1^ is contributed by asymmetric bending of a methyl group which may relate to esters and carbohydrate. The band around 1410 cm^−1^ may be assigned as the stretch vibrations of C-O band, which may be associated with glucoside. Around the peaks of 1375 and 1365 cm^−1^, bending vibration of methyl groups arises, which may come from esters compounds. Besides, the bands located at 945 and 933 cm^−1^ are results of sugar moieties vibrations related to glycoside. According to previous study [42], the band around 1750–1550 cm^−1^ may be related to iridoid glycosides. This also is coincident with the quantitative analysis (Table 2).

### 3.6. Rapid Prediction of Gentiopicroside Based on FTIR and HPLC Methods

**Figure 7:**
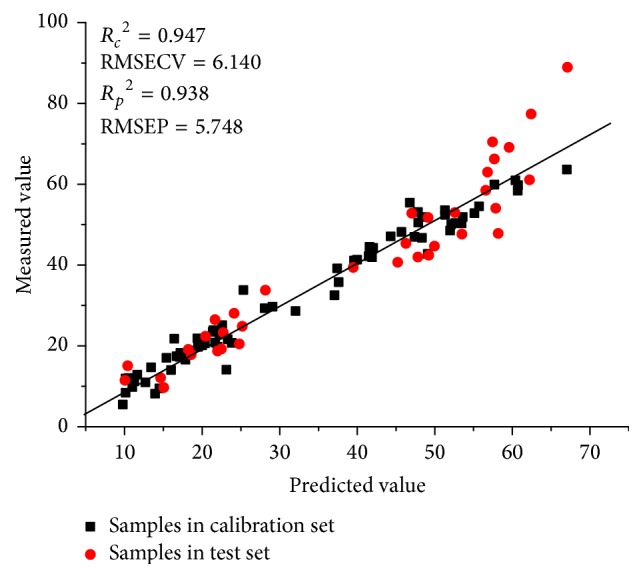
The analytical result of determination of gentiopicroside. (Description: the relationship between the predicted and measured concentrations of gentiopicroside is presented in this figure, based on PLSR technique.)

In Chinese Pharmacopoeia, the concentration of gentiopicroside is the standard to control the quality of* G. rigescens* with the threshold of 1.5% in weight [13]. To the best of our knowledge, the determination of gentiopicroside in* G. rigescens* has been developed using FTIR spectroscopy and PLSR algorithm in a previous study, with a RPD of 2.701 [43]. In this study, the results are presented in Table 3. The best PLSR model is established by pretreatments of MSC and 2D, with the *R*_*C*_^2^, RMSECV, *R*_*p*_^2^, RMSEP, and RPD of 0.947, 6.140, 0.938, 5.748, and 3.646. The relationship between the predicted and measured concentration is presented in Figure 7. By the selection of characteristic band, the performance of PLSR model for determination of gentiopicroside is improved, demonstrating that FTIR spectroscopy can provide a rapid and simple tool to control the quality of* G. rigescens*.

**Figure 8:**
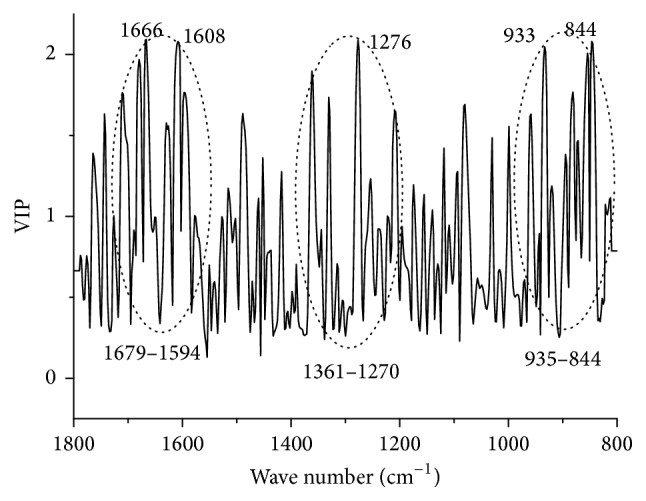
The VIP plot of PLSR model. (Description: this plot is used to identify the bands which are higher related to the determination of gentiopicroside in the regression model.)

The variable importance in the projection (VIP) is the criterion that explores the importance of variables in PLSR model [44]. In order to identify the bands which are higher related to the prediction, VIP plot is extracted in Figure 8. The bands around 1679–1594, 1361–1270, and 935–844 cm^−1^ are relevant to the determination of gentiopicroside. Particularly, peaks at 1666, 1608, 1276, 933, and 844 cm^−1^ make the most contributions to the prediction. The peak at 1666 cm^−1^ may be due to carbonyl group [17]. The peaks at 1610 and 1276 cm^−1^ may be results of terpene compounds, while the bands located at 933 and 844 cm^−1^ may be caused by vibrations of sugar moieties related to glycoside [42].

## 4. Conclusion

The current paper firstly reported a comprehensive evaluation of different parts and cultivation years of* G. rigescens* resource using HPLC and FTIR techniques. The accumulation of iridoid glycosides (loganic acid, swertiamarin, gentiopicroside, and sweroside) among different parts and cultivation years obviously changed. These variations can be effectively discriminated based on FTIR profile of 1800–800 cm^−1^ band in two-dimensional score plots. In addition, a satisfactory determination of gentiopicroside was also performed using FTIR spectroscopy and PLSR technique. As a consequence, results obtained from these complementary techniques can provide a systematic characterization for the quality control of this medicinal plant. Moreover, this study can contribute to the rational development and utilization of* G. rigescens* resources.

## Supplementary Material

The plant of Gentiana rigescens.

## Figures and Tables

**Table 1:** The calibration curves, *r*^2^, LOD, and LOQ of the standard compounds.

Analytes	Calibration curves	Linearity (*μ*g/mL)	*r* ^2^	LOD (*μ*g/mL)	LOQ (*μ*g/mL)
Loganic acid	*y* = 7115.1222*x* + 24.8007	4.4–800.0	0.99995	5.3	19.1
Swertiamarin	*y* = 7976.8679*x* + 25.1673	1.7–800.0	0.99991	4.1	13.8
Gentiopicroside	*y* = 5830.2818*x* + 164.9953	49.8–3600.0	0.99991	42.9	142.9
Sweroside	*y* = 4250.2396*x* − 2.0569	1.8–700.0	0.99996	7.1	23.5

**Table 2:** The variation of total iridoid glycosides according to different parts and cultivation ages.

*G. rigescens*	Root	Stem	Leaf	Flower
1 year old	55.71 ± 8.99^b^	24.67 ± 6.57^cd^	30.61 ± 8.68^c^	62.55 ± 5.87^ab^
2 years old	67.52 ± 16.90^a^	23.93 ± 12.74^cd^	21.36 ± 10.22^d^	56.43 ± 8.00^b^
3 years old	63.74 ± 8.53^ab^	28.92 ± 6.40^cd^	33.66 ± 13.80^c^	62.34 ± 10.74^b^

(*Notes*. Different superscript letters within the same row or column indicate significant differences at *p* ≤ 0.05 according to Duncan test.)

**Table 3:** The results for determination of gentiopicroside based on PLSR technique.

Wave number (cm^−1^)	Pretreatments	*R*_*c*_^2^	RMSECV	*R*_*p*_^2^	RMSEP	RPD
4000–400	No pretreatment	0.940	11.109	0.794	9.746	2.150
4000–400	MSC + 1 D	0.972	7.083	0.879	7.924	2.645
4000–400	MSC + 2 D	0.968	6.495	0.905	6.730	3.114

1800–800	No pretreatment	0.917	6.981	0.837	9.076	2.309
1800–800	MSC + 1 D	0.927	7.500	0.911	7.068	2.965
1800–800	MSC + 2 D	0.947	6.140	0.938	5.748	3.646
