# Supplementary figures and images for: Quantitative and Qualitative Characterization of Gentiana rigescens Franch (Gentianaceae) on Different Parts and Cultivations Years by HPLC and FTIR Spectroscopy
Source: J Anal Methods Chem. 2017 Jun 1;2017:3194146. doi: 10.1155/2017/3194146 (PMC5471563; doi:10.1155/2017/3194146)

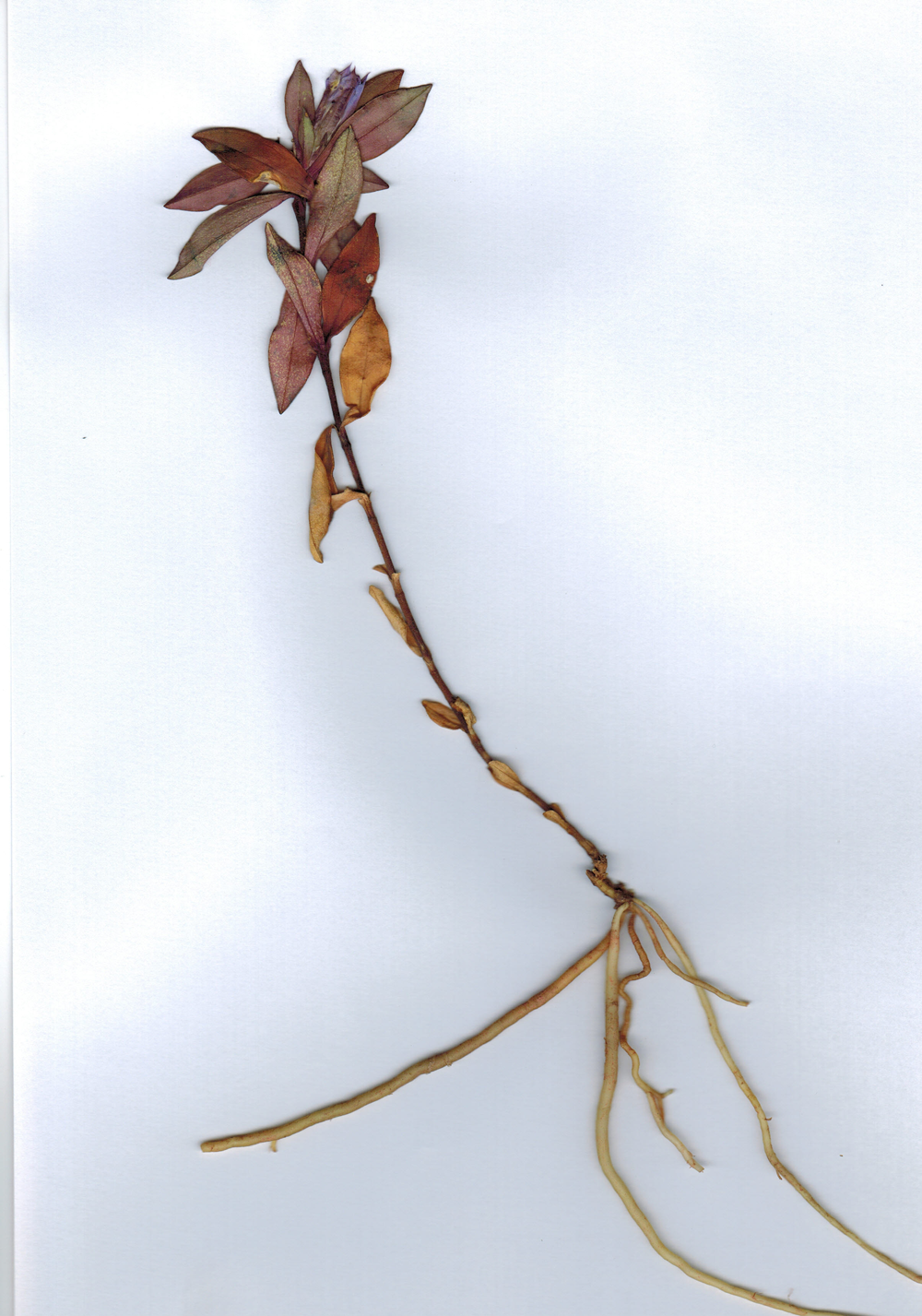

Supplement: Supplementary file 1 — The plant of Gentiana rigescens. [file 3194146.f1.tif]
